# Comparison of Imaging Characteristics of ^124^I PET for Determination of Optimal Energy Window on the Siemens Inveon PET

**DOI:** 10.1155/2016/3067123

**Published:** 2016-03-22

**Authors:** A Ram Yu, Hee-Joung Kim, Sang Moo Lim, Jin Su Kim

**Affiliations:** ^1^Laboratory Animal Center, OSONG Medical Innovation Foundation, Osong, Chungbuk 28160, Republic of Korea; ^2^Department of Radiological Science, College of Health Science, Yonsei University, Wonju 26493, Republic of Korea; ^3^Molecular Imaging Research Center, Korea Institute of Radiological and Medical Sciences, Seoul 01812, Republic of Korea; ^4^Korea Drug Development Platform Using Radio-Isotope (KDePRI), Seoul 01812, Republic of Korea; ^5^Radiological and Medico-Oncological Sciences, University of Science and Technology (UST), Seoul 01812, Republic of Korea

## Abstract

*Purpose.* ^124^I has a half-life of 4.2 days, which makes it suitable for imaging over several days over its uptake and washout phases. However, it has a low positron branching ratio (23%), because of prompt gamma coincidence due to high-energy *γ*-photons (602 to 1,691 keV), which are emitted in cascade with positrons.* Methods.* In this study, we investigated the optimal PET energy window for ^124^I PET based on image characteristics of reconstructed PET. Image characteristics such as nonuniformities, recovery coefficients (RCs), and the spillover ratios (SORs) of ^124^I were measured as described in NEMA NU 4-2008 standards.* Results.* The maximum and minimum prompt gamma coincidence fraction (PGF) were 33% and 2% in 350~800 and 400~590 keV, respectively. The difference between best and worst uniformity in the various energy windows was less than 1%. The lowest SORs of ^124^I were obtained at 350~750 keV in nonradioactive water compartment.* Conclusion.* Optimal energy window should be determined based on image characteristics. Our developed correction method would be useful for the correction of high-energy prompt gamma photon in ^124^I PET. In terms of the image quality of ^124^I PET, our findings indicate that an energy window of 350~750 keV would be optimal.

## 1. Introduction

PET is a widely used noninvasive diagnostic modality for imaging functional and biochemical phenomena* in vivo*. The imaging of glucose metabolism using ^18^F-FDG is a routinely used PET imaging technique. The imaging of the hypoxia, perfusion, and proliferation is also possible using commonly used PET radionuclides, such as ^11^C, ^13^N, ^15^O, and ^18^F [1, 2]. Recently, the use of ^124^I has increased, since it is useful for pretherapeutic PET dosimetry and for studying monoclonal antibody kinetics to predict ^131^I activity distributions [3–5]. ^124^I could be used to investigate lengthy biological processes due to its long half-life (4.2 days), which makes it suitable for imaging over several days during the biological uptake and washout phases of radiolabeled antibodies [6, 7]. Characteristics of ^124^I were compared with ^18^F in Table 1. ^124^I has a low positron branching ratio (23%). High-energy *γ*-photons (602–1,691 keV) are emitted in a cascade with positrons, and a low positron branching ratio leads to decrease sensitivity. If high-energy *γ*-photons are recorded within the energy window, a prompt gamma coincidence, due to high-energy *γ*-photons, generates false coincidence. In addition, prompt gamma coincidence contributed to the detection of spurious background activity [8, 9].

Furthermore, high-energy gammas are emitted in cascade with positrons. These lead to spurious coincidences and reduction of image quality [10, 11]. Although several research groups have studied the quantification and correction of prompt gamma coincidence for ^124^I PET [9, 10, 12–14], the quantification of ^124^I PET remains challenging. Because prompt gamma coincidence due to high-energy *γ*-photons was emitted in ^124^I PET, optimal PET acquisition settings were required to improve image quality. Other papers were studied count based evaluation and did not consider the prompt gamma coincidence correction [8, 10, 15]. So, they suggest use of relatively narrow energy window in order to exclude high-energy gamma prompt photons. Although the NECR was used to assess the image quality of PET, recently there was a report that SNR of reconstructed image cannot be predicted by the NECR especially on the PET image using iterative reconstruction method [16, 17]. Because raw data may not follow a Poisson distribution due to dead time, thus, the use of the NECR may be limited [16, 18].

In contrast, we proposed prompt gamma coincidence correction method in a previous study [19]; we could successfully correct the prompt gamma photon in ^124^I. Our developed method was applied to reconstructed image not count based NECR metric. Therefore, optimal energy window in ^124^I PET should be determined on reconstructed image. In this study, we assessed the image quality of reconstructed PET to determine the optimal energy window in ^124^I PET with various parameters on Siemens Inveon PET (Siemens Medical Solutions, USA) [20–22]. Image qualities such as nonuniformity, recovery coefficient, and spillover ratio were assessed according to NEMA NU 4-2008.

## 2. Materials and Methods

### 2.1. System Description

The Inveon system was preclinical system with high sensitivity and high resolution. The detector consists of 64 detector blocks with a 16.1 cm of ring diameter and 12.7 cm of axial FOV. The crystal size is 1.51 × 1.51 × 10 mm^3^ and the crystal pitch is 1.59 mm. The packing fraction is 92% [15, 20–22].

### 2.2. Correction of Prompt Gamma Coincidence of ^124^I

Recently, we developed the method for determination of prompt gamma coincidence fraction of ^124^I [19]. Briefly, the process of measurement of sensitivity and prompt gamma coincidence fraction were described as follows; to measure the prompt gamma coincidence fraction, we measured the sensitivities of ^124^I and ^18^F using NEMA NU 2 like sensitivity phantom (length: 12.7 cm) [23]. Source activities were 506 kBq for ^124^I and 673 kBq for ^18^F, respectively. Sensitivities were measured at the condition below 1% dead time loss. PET data was collected for 5 min using 1~5 aluminum sleeves of 1 mm thickness and different diameters. Intrinsic and background activities were measured for 1 hr and subtracted from total prompt counts within every energy window set. Branching ratio corrected sensitivities of ^124^I and ^18^F were also calculated. Sensitivities and prompt gamma coincidence fraction were calculated with various energy windows. Prompt gamma coincidence fractions (PGFs) were predicted using the following equation: 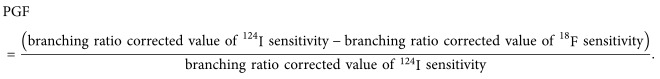
(1)


We determined prompt gamma coincidence fraction at various energy windows. The lower level discriminator (LLD) was fixed at 350 keV to minimize the effect of intrinsic radioactivity due to the presence of ^176^Lu [24, 25]. Because adjustment of the upper level discriminator (ULD) was influential for the assessment of prompt gamma coincidence due to the high-energy single *γ*-photons of ^124^I (602 to 1,691 keV), the ULD was increased in steps of 25 or 50 keV from 550 keV to 800 keV. Energy window of 400~590 keV was also included to compare the result with previous study [8]. To perform the prompt gamma coincidence correction [19], we derived the sinogram for the scatter component and then multiplied by a PGF. The prompt gamma coincidence corrected emission sinogram was then determined as follows:

(2) Prompt  gamma  coincidence-corrected  emission  sinogram=emission  sinogram−scatter  sinogram×PGF.

### 2.3. Image Quality

NECR is the metric based on measured PET count rate. Therefore, we assessed image quality on reconstructed ^124^I PET image to determine the optimal ^124^I PET energy window. Image qualities such as nonuniformity, recovery coefficient (RC), and spillover ratio (SOR) were measured according to NEMA NU 4-2008. The NEMA NU 4-2008 image quality phantom (length 50 mm, diameter 30 mm, and volume 20.7 mL) is consisted of three parts in cylindrical. Space of the center is uniform region (length 15 mm, diameter 30 mm) and this part means actual signal to noise of imaging equipment. The upper part of the uniform region is cold region that has two empty spaces (length 15 mm, inner diameter 8 mm, and outer diameter 10 mm). One space fills air and the other space fills nonradioactive water. Although both cylinders are nonradioactive, scattered photons, nonzero positron range, and random or other effects may cause the reconstructed images to display activity in these compartments. So measurement of spillover ratio from cold region displays accuracy of scatter correction. Bottom of the cylinder (length 20 mm, diameter 30 mm) has five fillable rods with diameters of 1, 2, 3, 4, and 5 mm and center of each rod is 7 mm from the cylinder axis. This part serves recovery coefficient.

According to the NEMA NU 4-2008 guideline, 20 min of scan time and 3.7 MBq of ^18^F were needed. Because ^124^I has a lower positron branching ratio compared to that of ^18^F, longer scan time and higher activity of ^124^I were needed to avoid the bias due to the difference of positron number when image quality was compared. In the study of Disselhorst and coworkers, a corrected scan time of 4700 s and activity of 14.4 MBq were used for ^124^I PET, because equal positron numbers were needed for proper comparison as the branching ratios of ^124^I and ^18^F were different [11]. In our present study, we also used 4700 s of PET scan time and 14.4 MBq of ^124^I to maintain the same level of positron numbers when image characteristics of ^124^I were investigated. The image quality phantom was placed in the center of the scanner FOV and PET data was acquired at various energy windows settings to determine the optimal PET energy window. The lower level discriminator was fixed at 350 keV, and the higher level discriminator was increased in steps of 25 or 50 keV steps from 550 keV to 800 keV. The timing window was set to 3.432 nsec. 3D list mode PET data were sorted into sinogram using FORE and reconstructed using 2D FBP with a ramp filter. The pixel size of reconstructed images was 0.776 × 0.776 mm^2^. Attenuation correction was performed using ^57^Co point source [26]. Normalization, scatter correction (SC), prompt gamma coincidence correction, and dead time correction were also applied.

#### 2.3.1. Nonuniformity

To measure nonuniformity, a volume of interest (VOI) (length 10 mm, diameter 22.5 mm) was drawn at the center of the uniform region. The values of the means and standard deviations (SD) in this VOI were measured. Nonuniformity (NU) was expressed as percentage SD (%SD: standard deviation divided by mean multiplied by 100%).

#### 2.3.2. Recovery Coefficient

The five radioactive source fillable rods (diameters 1, 2, 3, 4, and 5 mm) in the bottom of the cylinder (length 20 mm, diameter 30 mm) were used to determine RCs. To determine the RCs, circular ROI of twice the rod diameter was drawn around each rod. Maximum values and SDs were measured for each profile. RC was defined as the ratio between the measured maximum values in rods and the mean value in the uniform area. %SD of RCs were calculated using the following equation:

(3) %SDRC=100×SDline  profileMeanline  profile2+SDuniform  regionMeanuniform  region2.

#### 2.3.3. Spillover Ratio

The upper part of the uniform region was a cold region consisting of two empty spaces (length 15 mm, inner diameter 8 mm, and outer diameter 10 mm). One empty space was for air and the other space was for nonradioactive water. To calculate the SOR, two cylindrical VOIs (length 7.5 mm, diameter 4 mm) were drawn in the air and nonradioactive water filled compartments. The half size of the water or nonradioactive compartments was used to minimize the effect of the longer positron range of ^124^I in ROI analysis [11]. SOR was defined as the ratio between the mean value of a cold cylinder and the mean value of the uniform area. The effect of prompt gamma coincidence due to high-energy *γ*-photons (602 keV to 1,691 keV) emitted from ^124^I was assessed in terms of SOR for different PET acquisition energy windows.

### 2.4. Noise Equivalent Count Rate

In another previous study, an optimal energy window for ^124^I small animal imaging was determined using NECR [8]. In our present study, we also calculated NECRs using Monte Carlo simulation [27] to compare the result of image quality assessment on reconstructed image.

Inveon consists of 16 modules providing 80 full crystal rings with an axial length of 127 mm and a ring diameter of 161 mm. Size and pitch of crystal are 1.51 × 1.51 × 10 mm^3^ and 1.59 mm. Energy resolution was 14.5% and coincidence window was set to 3.432 ns [20]. NEMA NU 4 mouse phantom (diameter = 2.5 cm, length = 7 cm) was placed at the center. ^124^I source simulated all the physical process during emission and interaction of positrons. NECR was then calculated as the following equation:

(4) $$\begin{array}{c} NECR=\frac{T^{2}}{T+S+2fR+fPGF}, \end{array}$$

where *T*, *S*, *R*, PGF, and *f* are prompt gamma coincidence corrected true, scatter, random, and prompt gamma coincidence fraction and average fraction of the projection taken up by the object. Simulation for NECR was repeated under various energy windows. The lower level discriminator was fixed at 350 keV, and the higher level energy discriminator was increased in steps of 25 keV from 550 keV to 800 keV. Energy window of 400~590 keV was also included. Source activity was 1 MBq to 50 MBq for measurement of NECR.

## 3. Results

### 3.1. Prompt Gamma Coincidence Fraction of ^124^I

The branching ratio uncorrected ^124^I sensitivities in representative energy window were 1.57, 1.78, 2.04, 2.26, and 1.31% within the energy window of 350~550, 350~600, 350~650, 350~750, and 400~590 keV, respectively. The branching ratio uncorrected ^18^F sensitivities were 6.44, 6.51, 6.54, 6.61, and 5.43% within the energy window of 350~550, 350~600, 350~650, 350~750, and 400~590 keV, respectively. The branching ratio corrected sensitivities were 9.83% for ^124^I and 6.81% for ^18^F within an energy window of 350~750 keV and timing window of 3.432 ns. The difference between “branching ratio corrected” and “branching ratio uncorrected” sensitivities was the portion of prompt gamma coincidence fraction. The PGF was 31% at 350~750 keV. The PGFs were presented in Table 2. The maximum and minimum PGF were 33% and 2% in 350~800 and 400~590 keV, respectively.

### 3.2. Image Quality

#### 3.2.1. Nonuniformity

**Figure 1:**
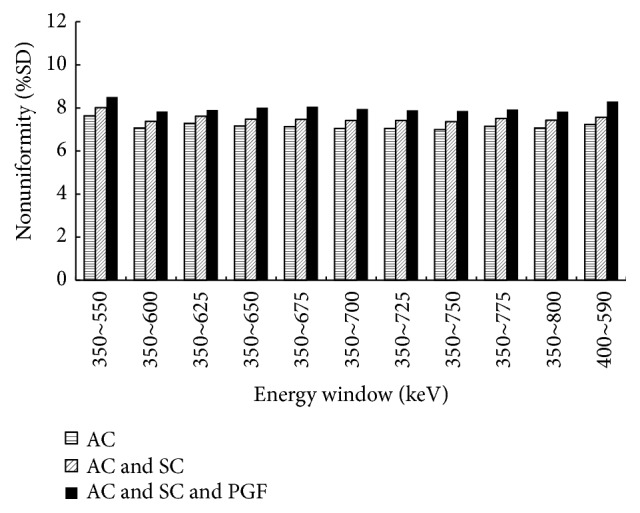
Nonuniformity (%SD) in the uniform region of the NEMA NU 4 image quality phantom.

The nonuniformity of the NEMA NU 4 image quality phantom for ^124^I was shown in Figure 1. The highest and the lowest nonuniformities were 7.63% and 6.99% within energy windows of 350~550 and 350~750 keV, respectively. The difference of nonuniformity with the various energy windows was <1%. When SC and PGF were applied, nonuniformity was slightly worse. The mean difference was 0.35 and 0.84 percentage point when applying SC and PGF correction, respectively, compared to applying only AC.

#### 3.2.2. RC

The RC and %SD of RC values of the different rods were shown in Table 3. For ^124^I PET, 1 mm sized rod was not discernible on the image. Therefore the RC for a 1 mm sized rod was not calculated. The representative values of RC within an energy window of 350~750 keV were as follows. RCs were 0.27, 0.41, 0.54, and 0.67 for 2, 3, 4, and 5 mm rods, respectively.

#### 3.2.3. SOR

SORs in the air and nonradioactive water compartments were shown in Table 4 with different energy windows. The lowest SOR value was obtained within an energy window of 350~700 and 350~750 keV in air and water, respectively. The lowest SOR values in air and water compartments were −6.47% and 0.26% at 350~700 and 350~750 keV, respectively. Negative value of SOR after scatter correction was due to bias scaling of single scatter simulation algorithm which was reported in Disselhorst and colleagues' study [11]. The highest SOR value was obtained within an energy window of 350~650 keV in both air and water. When SC was compared with AC, SOR values were decreased by roughly 4.28 percentage point in air and by 6.74 percentage point in water. When PGF was compared with AC, SOR values were decreased by roughly 7.66 percentage point in air and by 12.79 percentage point in water.

Considering the actual mouse experimental conditions, the weighted SOR (wSOR) was calculated using the following equation:

(5) $$\begin{array}{c} wSOR=\sqrt{f_{air}\cdot {{SOR}_{air}^{}}^{2}+f_{water}\cdot {{SOR}_{water}^{}}^{2}}. \end{array}$$

The weighting factor of *f*_air_ and *f*_water_ represents the ratio of the volume occupied by the air and water, respectively, in mouse whole body. The value of *f*_air_ was the ratio of lung volume in mouse carcass which was calculated using organ density and mass for mouse model (*f*_air_: 0.027, *f*_water_: 0.973) [28]. Considering the actual mouse experimental conditions, air (lung)/water fractions were determined. The minimum value of the wSOR was assumed to be the desired point, and the corresponding energy window was the optimal energy window. The lowest wSOR value was obtained within an energy window of 350~750 keV for reconstructed images when AC, SC, and PGF corrections were applied (Table 4).

### 3.3. Noise Equivalent Count Rate

**Figure 2:**
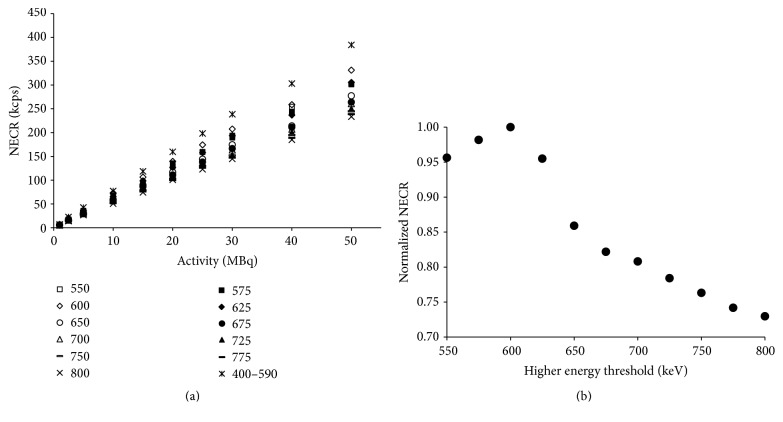
NECR curve for different energy windows (a) at the lower threshold was 350 keV in all cases except 400~590 keV. Normalized NECR variation according to ULD (b).

Figure 2(a) shows the NECR curve in different energy windows. The highest NECR was obtained at 400~590 keV. The 2nd highest NECR was found at 350~600 keV and the NECR was decreased as the ULD was increased (Figure 2).  Figure 2(b) showed which energy window has the maximum of the NECR. The NECR was calculated with 5 MBq of ^124^I, the actual activity of injecting the mouse. The LLD was first fixed at 350 keV, and the ULD was increased in 25 keV steps starting at 550 keV until 800 keV. The normalized NECR curves were generated with optimized energy windows. Individual NECR was normalized with the value of NECR at 350~600 keV. Normalized value of NECR with varying ULD was plotted in Figure 2(b).

## 4. Discussion

In this study, we measured the PET sensitivity and image quality parameters of ^124^I in order to identify an optimal energy window for ^124^I imaging. We can find that PGF was increased with wider energy windows (Table 2). PGF at 350~550 and 400~590 keV was almost zero. This result is consistent with the other paper [8].

Nonuniformity was increased within narrower energy window. The highest and the lowest nonuniformities were 7.63% and 6.99% within energy windows of 350~550 and 350~750 keV, respectively. The difference between the lowest and the highest nonuniformities with various energy windows was <0.6 percentage point. When SC and PGF were performed, nonuniformity became slightly worsened due to reduced measured count within corresponding energy windows. This tendency for increase of nonuniformity after SC has been previously reported [11]. The difference of nonuniformity was 0.35 percentage point whether the scatter correction is performed or not. When PGF was performed, the difference of nonuniformity was larger than SC (0.84 percentage point).

The RCs of the 4 different rods were shown in Table 3. In this study, since a 1 mm diameter rod was not discernible by ^124^I PET, we could not calculate the RC of a 1 mm diameter for ^124^I. This was due to the properties of limit resolution of ^124^I. A low RC value means low detectability in small hot regions (<1 mm diameter) for ^124^I PET due to higher positron range than ^18^F. According to Bao et al.'s report, the RCs of ^18^F were 0.17, 0.48, 0.72, 0.84, and 0.93 mm for 1, 2, 3, 4, and 5 mm rods, respectively [22]. The difference of RCs between ^124^I and ^18^F was about 30% compared with Bao et al.'s paper. These were associated with the reduced spatial resolution. The FWHMs (full width at half maximum) were reported to be 2.38 mm and 1.81 mm for ^124^I and ^18^F, respectively (about 30% difference) [11]. Regarding the effect of the energy window, RCs were not significantly different within the energy windows (*p* > 0.05). RC was dependent on the spatial resolution. %SD of RC was increased for wider energy windows. When SC and PGF were applied, RCs were not significantly changed (*p* > 0.05). This was well accordant with a previous study [11].

SORs in the nonradioactive water and air compartments were shown in Table 4. The lowest SOR value was obtained within an energy window of 350~700 and 350~750 keV in air and water, respectively. The highest SOR value was obtained within an energy window of 350~650 keV in both air and water. After PGF correction, SORs were improved by 7.66 percentage point and 12.79 percentage point at air and nonradioactive water compared to the value of SOR after AC. In this present study, we compared multiple parameters for the assessment of image quality for determination of optimal PET energy window of ^124^I. Because sensitivity and nonuniformity simply depend on PET count statistics, RC was not changed with different energy windows. Therefore, assessment of SOR would be more crucial for determination of optimal PET acquisition window. In particular, for ^124^I PET, single gamma photons also should be considered for the analysis of SOR. The SOR was improved after single gamma photon correction; our proposed correction method was reasonably suitable for correction of single gamma photon. Although SOR in water included the effects of scatter and positron range, SOR in air reflected only scattered photons; because the positron range in air was more than 1 m, almost no annihilations occurred in air [11]. Thus, the value of SOR in the water was more important than in air. For ^124^I PET, image quality was found to be affected by SOR for various energy windows and prompt gamma photon correction. We calculated wSOR to determine the optimal energy window considering the composition ratio of air and water. The best value of wSOR was obtained with an energy window of 350~750 keV.

The NECR has been commonly proposed to find optimal parameters of PET scanners. Therefore, in another previous study, optimal energy windows for ^124^I small animal imaging with the Inveon PET system were determined based on NECR [8]. In our NECR result, the optimized ULD was determined to be 600 keV, among our experimental design of energy window setting. This is the most similar ULD proposed in other experiments (590 keV) [8]. Additionally, the highest NECR was obtained at 400~590 keV. This result was in agreement with the other study [8].

Optimal energy windows based on NECR were determined to be 350~600 keV in present study and 400~590 keV in the other study. The values of wSOR were 1.43 and 1.67 with an energy window of 350~600 keV and 400~590 keV, respectively. The values of wSOR with an energy window of 350~600 keV and 400~590 keV were not lowest when energy window was determined based on NECR. The lowest wSOR values were 0.95 with an energy window of 350~750 keV. This was because NECR did not account for possible count rate bias such as the systematic mispositioning of data because of spatial pile-up effects [29]. This phenomenon was more prominent for ^124^I due to the emissions of high-energy gamma photons. Therefore, evaluation of image quality was necessary to choose the appropriate energy window. Optimal energy window was determined by considering the analysis of SOR. Although the PGF was increased with wider energy windows, the best SOR was showed in 350~750 keV. As applying the prompt gamma correction, conditions that guarantee the count rate to some extent were optimal energy window. In this study to determine the energy window, our interest is not count based evaluation but reconstructed image quality based evaluation. We have proposed a relatively wide energy window; it means the proposed correction method considering PGF was useful to quantify the ^124^I PET. Therefore, there was a big difference between our proposed method (prompt gamma correction method) and NECR metric. According to our proposed method, energy window “350–750 keV” was best energy window. Although this energy window was wider, prompt gamma coincidence could be corrected using our proposed prompt gamma correction method and this result was assessed using image quality from the reconstructed PET data. Therefore, we could use wider energy window for ^124^I.

## 5. Conclusion

The present study described the image characteristics to suggest an energy window of ^124^I. Optimal energy window should be determined based on image characteristics and the SOR was important factor to determine the optimal energy window in ^124^I PET. Our developed prompt gamma correction method would be useful for the quantification of ^124^I PET.

## Figures and Tables

**Table 1:** Characteristics of ^124^I in comparison with ^18^F.

	^124^I	^18^F
Half-life	4.18 day	109.74 min

Max. positron energy (keV)	2,138	635

Branching ratio (%)	23	97

Gamma energy (keV)	511 (23%) 602 (60%) 722 (10%) 1,691 (11%)	511 (97%)

**Table 2:** Prompt gamma coincidence fraction (PGF) at different energy window settings.

Energy window (keV)	PGF
350~550	0.03
350~600	0.13
350~625	0.19
350~650	0.24
350~675	0.26
350~700	0.27
350~725	0.29
350~750	0.31
350~775	0.32
350~800	0.33
400~590	0.02

**Table 3:** Recovery coefficients and %SD values for 5 different rods.

Rod diameter^*∗*^ (mm)	2		3		4		5	
	RC^†^	%SD^‡^	RC	%SD	RC	%SD	RC	%SD
FBP								
350~550	0.23	32.90	0.36	44.09	0.53	50.20	0.64	65.26
350~600	0.24	29.89	0.39	40.54	0.50	46.35	0.62	63.38
350~625	0.27	33.43	0.40	45.78	0.52	47.42	0.62	62.90
350~650	0.27	31.06	0.38	42.24	0.49	46.74	0.63	62.11
350~675	0.22	27.41	0.38	42.26	0.60	45.78	0.60	61.49
350~700	0.25	28.95	0.40	41.27	0.57	46.76	0.67	61.51
350~725	0.26	30.60	0.37	40.65	0.52	47.11	0.65	62.01
350~750	0.27	31.20	0.41	44.30	0.54	46.57	0.67	61.64
350~775	0.24	29.34	0.41	41.95	0.57	47.07	0.65	62.14
350~800	0.23	25.71	0.41	41.56	0.55	46.94	0.68	62.98
400~590	0.24	32.66	0.37	43.36	0.49	49.37	0.66	66.94

^*∗*^For ^124^I PET, RC could not be calculated for a 1 mm rod size, because this rod was not discernible by ^124^I PET.

^†^RC: recovery coefficient.

^‡^%SD: percent standard deviation.

**Table 4:** Spillover ratio in air and water compartments with attenuation, scatter, and single gamma photon correction applied.

Energy window	Air	Water	wSOR^‡^
350~550	−5.03	1.01	1.30
350~600	−4.33	1.26	1.43
350~625	−3.40	1.80	1.86
350~650	−2.68	2.87	2.86
350~675	−4.14	2.15	2.22
350~700	−6.47	0.79	1.32
350~725	−5.85	0.39	1.03
350~750	−5.54	0.26	0.95
350~775	−5.77	1.58	1.83
350~800	−5.41	2.36	2.49
400~590	−5.75	1.40	1.67

Mean difference^*∗*^	4.28	6.74	N/A^§^
Mean difference^†^	7.66	12.79	N/A

^*∗*^Mean difference: mean difference of spillover ratio between attenuation correction and attenuation and scatter correction.

^†^Mean difference: mean difference of spillover ratio between attenuation correction and attenuation and scatter and single gamma photon correction.

^‡^Weighted SOR (wSOR).

^§^Not applicable.
